# Oxalic acid and sclerotial differentiation of *Polyporus umbellatus*

**DOI:** 10.1038/srep10759

**Published:** 2015-06-01

**Authors:** Yong-Mei Xing, Wan-Qiang Yin, Meng-Meng Liu, Chun-Lan Wang, Shun-Xing Guo

**Affiliations:** 1 Key Laboratory of Bioactive Substances and Resource Utilization of Chinese Herbal Medicine, Ministry of Education, Institute of Medicinal Plant Development, Chinese Academy of Medical Sciences & Peking Union Medical College, No. 151, Malianwa North Road, Haidian District, Beijing, P. R. China 100193; 2 School of Biological Engineering, Tianjin University of Science & Technology, No.29, 13^th^ Avenue, Tianjin Economic and Technological Development Area (TEDA), Tianjin, China 300457

## Abstract

The present investigation aimed to uncover the effects of exogenous oxalic acid during the sclerotial formation of *Polyporus umbellatus*, with an emphasis on determining the content of the endogenic oxalic acid in the fungus. To this end, the oxalic acid content of the vegetative mycelia, sclerotia, culture mediums and sclerotial exudate were measured using High Performance Liquid Chromatography (HPLC). Furthermore, the lipid peroxidation was estimated by detecting thiobarbituric bituric acid reactive substances (TBARS). The results showed that the exogenous oxalic acid caused a delay in sclerotial differentiation (of up to 9 or more days), suppressed the sclerotial biomass and decreased the lipid peroxidation significantly in a concentration-dependent manner. Oxalic acid was found at very low levels in the mycelia and the maltose medium, whereas it was found at high levels in the mycelia and sucrose medium. After sclerotial differentiation, oxalic acid accumulated at high levels in both the sclerotia and the sclerotial exudate. Oxalic acid was therefore found to inhibit *P. umbellatus* sclerotial formation.

Oxalic acid is one of the most common constituents of plants^1^ and a wide variety of fungi^2^. It is known to play different roles in different organisms^3^,^4^. For example, Basidiomycetes can produce and utilize oxalic acid for lignin degradation^5^. Then, oxalic acid accumulation lowers the ambient pH, favouring *Sclerotinia sclerotium* sclerotial development^6^,^7^. Recently, an increasing number of researchers have become interested in systemic resistance and antioxidant systems after oxalic acid application^4^. For example, Kayashima and Katayama reported that oxalic acid, which is superior to malic acid, is a natural antioxidant, with the power to suppress lipid peroxidation in rat liver microsomes and in rat brain homogenate^8^. Furthermore, oxalic acid treatment has been found to delay mango ripening and significantly inhibit ethylene production^9^. Exogenous oxalic acid can decrease the production of active oxygen species and increase the activities of antioxidant enzymes in the peach fruit during storage^4^. It has also been reported that Sclerotinia (via oxalic acid) produces a reducing environment that suppress host defence responses, including oxidative stress^10^,^11^.

The sclerotium of *Polyporus umbellatus*, a valuable medicinal fungus, can be used as a diuretic agent. It has been used as an antidote in traditional Chinese medicine for many years, and its fruit body is edible and delicious. It has been reported that maltose and fructose are suitable carbon sources to induce *P. umbellatus* sclerotia directly from hyphae to sclerotia under artificial conditions^12^,^13^,^14^. Three distinct stages, including sclerotial intiation (SI), development (SD) and maturation (SM), occurred during *P. umbellatus* sclerotial differentiation^14^,^15^. Sclerotial exudate appeared in the SD and SM stages of sclerotia induced by maltose medium^15^. Previous studies have shown that *P. umbellatus* sclerotial development is closely associated with a high oxidative state, and H_2_O_2_ accumulation was observed in cell walls or around the organelle membranes of the mycelial cells using transmission electronic microscopy (TEM)^16^.

It is known that in response to oxidative stress, enzymatic and non-enzymatic systems play an important role in preventing significant oxidation-induced damage^17^,^18^. Thus, endogenous antioxidants such as β-carotene^19^,^20^,^21^,^22^, ascorbic acid^19^,^20^,^23^,^24^, ascorbate and erythroascorbate^25^ have been receiving much research attention. It remains unclear whether endogenous oxalic acid serves as an antioxidant in fungal growth and development.

Many calcium oxalate crystals were found in the natural sclerotia of *P. umbellatus*. Previously, oxalate crystals were observed during the cultivation of sclerotia under artificial conditions using scanning electronic microscopy (SEM)^26^, begging the question: what roles does the endogenous oxalic acid play during *P. umbellatus* mycelial growth and sclerotial formation? This question sparked our interest in determining the endogenous oxalic acid levels in different cultivation mediums, mycelia, sclerotia and the sclerotial exudate of *P. umbellatus*. Elucidating the possible effects of exogenous oxalic acid on the physiological and biochemical behaviour of *P. umbellatus* sclerotial differentiation in this context is also important. The results from this study will help further our understanding of the biological process of *P. umbellatus* sclerotial transformation.

## Methods

### Fungal strains

The *P. umbellatus* strain was isolated from Guxian (Shanxi Province of China) and incubated on solid wheat bran medium at 25 °C in the dark. After inoculation, the Petri dishes were placed at 25 °C in the dark. The fungus was identified in a previous study through the molecular biological analysis of the internal transcribed region (ITS) of the 5.8S rDNA^27^.

### *P. umbellatus* cultivated in different culture mediums

The maltose medium consisted of maltose 20 g L^−1^, peptone 4 g L^−1^, K_2_HPO_4_∙3H_2_O 1 g L^−1^, KH_2_PO_4_ 0.5 g L^−1^, MgSO_4_∙7H_2_O 0.5 g L^−1^, vitamin B1 0.05 g L^−1^ and agar 12 g L^−1^, as described in a previous study^14^. The sucrose medium contained 20 g L^−1^ of sucrose; all other components were the same as in the maltose medium.

### Chemicals and materials

All reagents and chemicals were of analytical grade. They were obtained from the Chinese National Institutes for Food and Drug Control and were used without any further purification.

### Effect of exogenous oxalic acid on *P. umbellatus* sclerotial differentiation

Before inoculation, different concentrations of the exogenous sterile oxalic acid were added to the sterilized growth medium through a 0.22-μm Millipore filter. The final concentrations of oxalic acid in the maltose medium were 0 mg ml^−1^, 0.5 mg ml^−1^, 1.0 mg ml^−1^, 5.0 mg ml^−1^ and 10.0 mg ml^−1^. The group without oxalic acid (0 mg ml^−1^) was used as the control group in these experiments. The sclerotial differentiation time, which was expressed as the sclerotial initiation (SI) appearance time and sclerotial formation time, was observed during the cultivation period after oxalic acid was added. After cultivation for 70 days, the sclerotia were separated, and their fresh weight was measured. The experiment was repeated three times, with 30 replicates in each group.

### Lipid peroxidation assay

The levels of the lipid peroxidation products in the mycelia and sclerotia were detected in the maltose and sucrose medium. The mycelia in the different media were separated gently from the fungal colonies. Subsequently, the samples were frozen in liquid nitrogen and ground in a porcelain mortar. Then, 0.1 g of powder was weighed and suspended in 4 ml 100 μM ethylenediaminetetraacetic acid (EDTA, disodium salt) solution and homogenized on ice. The homogenate was then centrifuged at 12000 *g* for 10 min. The supernatant was used for lipid peroxidation analysis by a modification of the TBA method^28^. Then, 600 μl of the supernatant was mixed with 200 μl 15% (w/v) TCA, 400 μl 1% (w/v) 2-thiobarbituric acid (TBA), and 6 μl 2% (w/v) butylated hydroxyanisol (BHA, dissolved in absolute ethanol). BHA was used as a lipid antioxidant to prevent artificial lipid peroxidation during the assay. The mixture was incubated at 100 °C for 30 min, then centrifuged at 12000 *g* for 10 min. The absorbance of the supernatant was measured at 535 nm and 600 nm against a blank sample of 600 μl 100 μM EDTA. The absorbance difference (A535-A600) was converted to the TBA reaction substance (TBARS) content, using the extinction coefficient for TBARS (1.56 × 10^5^ M^−1^ cm^−1^). Lipid peroxidation was expressed as nmol TBARS /mg protein. The protein concentration was assayed by a modification of a Coomassie Brilliant Blue (CBB)-based method^29^. Then, 0.1 ml of the supernatant was mixed with 1 ml of CCB-G250 reagent. After 5 min of incubation at room temperature, its absorbance was measured at 595 nm against a blank sample (using 100 μl 100 μM EDTA in place of the sample). The absorbance was converted into the protein content using a bovine serum albumin standard curve (0-100 μg ml^−1^), according to methods detailed in a previous study^15^.

### Effects of exogenous oxalic acid on the TBARS content of the mycelia of *P. umbellatus*.

The TBARS content of the mycelia was measured in the different periods of cultivation after different concentrations of oxalic acid were added to the maltose medium. The final concentrations of oxalic acid in the maltose medium were 0 mg ml^−1^, 0.5 mg ml^−1^, 1.0 mg ml^−1^, and 5.0 mg ml^−1^. The medium with no oxalic acid was administered to the control group. The experiment was repeated three times, with 30 replicates in each group.

### Determination of the endogenic oxalic acid content in *P. umbellatus*

Prior to measuring the oxalic acid content, a standard curve was generated with 100 μg/ml oxalic acid solution as a chemical reference substance. For the oxalic acid examination, a Synergi column (80 Å 4 μM C-18 250 × 4.6 mm, Waters, Milford, MA, USA) connected on-line with a Waters PAD-2996 photodiode array detector (set at 203 nm) at 30 °C was used. Aqueous oxalic acid was prepared in the concentration range of 10 μg ml^−1^ to 100 μg ml^−1^. Oxalic acid was extracted from the mycelia and sclerotia by the following method. Mycelia and sclerotia were shaved separately and gently from the colonies in petri dishes. They were then frozen in liquid nitrogen and ground into powder in a porcelain mortar. The total sample dry weight was determined by drying a small amount of the resulting powder at 60 °C for 2 h. The remaining powder was put into a 7 ml centrifuge tube, suspended in 3 ml of deionized water, extracted for 30 min by ultrasound, and then centrifuged at 12000 *g* for 10 min. The supernatant was collected. Then, 0.1 ml of a CaCl_2_ (500 mM) solution with 3 ml of absolute ethyl alcohol were added to the supernatant, which was then stored at 4 °C for 3 h. The samples were then centrifuged at 12000 *g* for 10 min. Subsequently, 0.5 ml of the H_2_SO_4_ (0.1 M) and 2 ml of the deionized water were added to the sediment. The resulting mixture was centrifuged at 12000 *g* for 10 min. The supernatant was stored at −80 °C prior to HPLC analysis. Oxalic acid was extracted from the culture medium using a method similar to that described above. After the fungal colonies were removed, the culture medium was ground in a porcelain mortar, then added to a 7 ml-centrifuge tube and weighed. The following procedure was the same as mentioned above. The samples were also stored at −80 °C before HPLC analysis. The Sclerotia exudate was collected into 1.5 ml-centrifuge tube with Pasteur pipettes, stored at −80 °C and diluted with deionized water before HPLC determination. For the oxalic acid determination, 10 μl of injection volumes were used for the HPLC analysis. Oxalic acid was chromatographed in a mobile phase containing 5 mM TBA and 0.01 M KH_2_PO_4_ (the pH was adjusted to 2.0 with the addition of phosphoric acid). The mobile phase was filtered through a 0.45 μm filter membrane before use^30^. The current velocity was 1 ml min^−1^. Oxalic acid from the samples was identified against the pure oxalic acid, and its concentration was determined by HPLC. Its concentration in the mycelia or sclerotia was expressed in mg g^−1^ dry weight, and the concentration of oxalic acid in the culture medium was denoted as μg g^−1^ fresh weight. The content of oxalic acid in the exudate was represented in mg ml^−1^.

### Data analysis

The data were analysed with a T-test or one-way ANOVA, and all statistical analyses were performed using SPSS 11.0 (SPSS, Chicago, IL, USA). Data were presented as the means ± SD from at least three independent experiments. *p* values < 0.05 were considered significant.

## Results

### The effects of exogenous oxalic acid on sclerotial formation

After cultivation for 70 days, no sclerotia formed in the groups treated with high concentrations of oxalic acid (5.0 mg ml^−1^ and 10.0 mg ml^−1^). Mycelia treated with low concentrations of oxalic acid (0.5 mg ml^−1^ and 1.0 mg ml^−1^) formed sclerotia; however, the sclerotia biomass was only 26% and 17% of that of the control group without oxalic acid. Furthermore, the time of SI appearance in the oxalic acid-supplemented groups was postponed to 24 days and 28 days after cultivation compared to 15 days after cultivation for the group without oxalic acid (Table 1). Exogenous oxalic acid caused a proportional inhibition in a concentration-dependent manner in *P. umbellatus* sclerotial differentiation.

### Lipid peroxidation products in mycelia cultivated in different mediums

**Figure 1:**
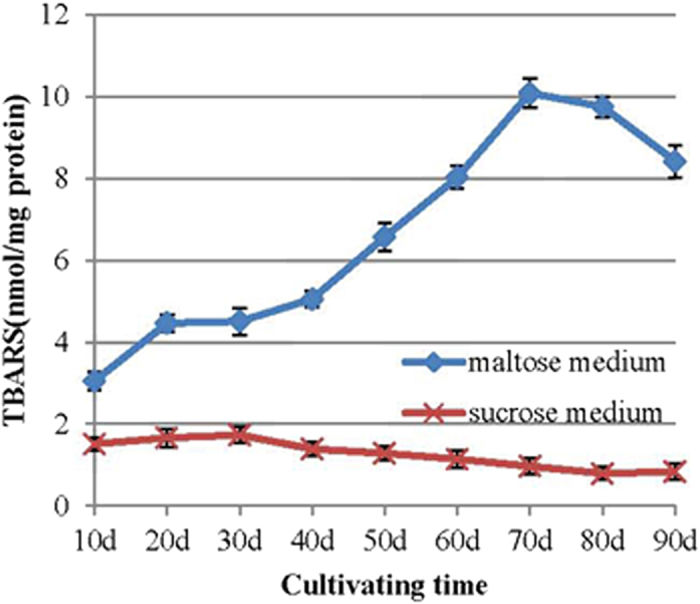
TBARS levels of the mycelia in the maltose and sucrose media. The independent T-test was used to analyse the data of the two groups for each cultivation phase. Mean values ± SD or representatives of at least three independent experiments are shown (n = 30).

The TBARS concentration in the mycelia of *P. umbellatus* cultivated in the maltose medium was much higher than that of *P. umbellatus* cultivated in the sucrose medium (*p *< 0.05) (Fig. 1). The concentration increased before the SI stage (20 days after inoculation), then slowly increased during the SD stage (30 or 40 days after cultivation). Then, the TBARS content increased sharply, reaching its maximum during the SM stage (70 days after cultivation) and then decreasing gradually. The concentration of TBARS in the mycelia in the maltose medium reached 10.08 nmol/ mg protein, which was 10.39 times the concentration reached in the sucrose medium. The TBARS content of the mycelia in sucrose medium remained at low levels (below 2 nmol/ mg protein) throughout the cultivation time (Fig. 1).

### The effects of exogenous oxalic acid on the TBARS content of the mycelia of *P. umbellatus*.

**Figure 2:**
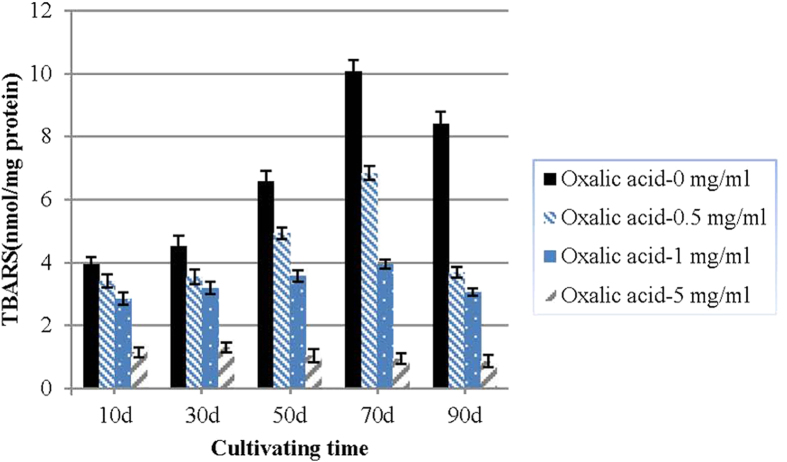
Effects of exogenous oxalic acid on the TBARS content of the mycelia of *P. umbellatus*. One-way ANOVA was used to analyse the data of the two groups for each cultivation phase. The data presented represent the means ± SD from three independent experiments, with 30 replicates in each group.

Exogenous oxalic acid caused a concentration-dependent reduction in lipid peroxidation. The reduced TBARS content was accompanied by an increased oxalic acid concentration. At the time of the SM stage, the TBARS content of the groups without oxalic acid was 1.47-fold, 2.55-fold and 10.61-fold of the groups with 0.5 mg ml^−1^,1.0 mg ml^−1^ and 5.0 mg ml^−1^ oxalic acid, respectively (Fig. 2).

### Determination of the endogenic oxalic acid during *P. umbellatus* growth and development

**Figure 3:**
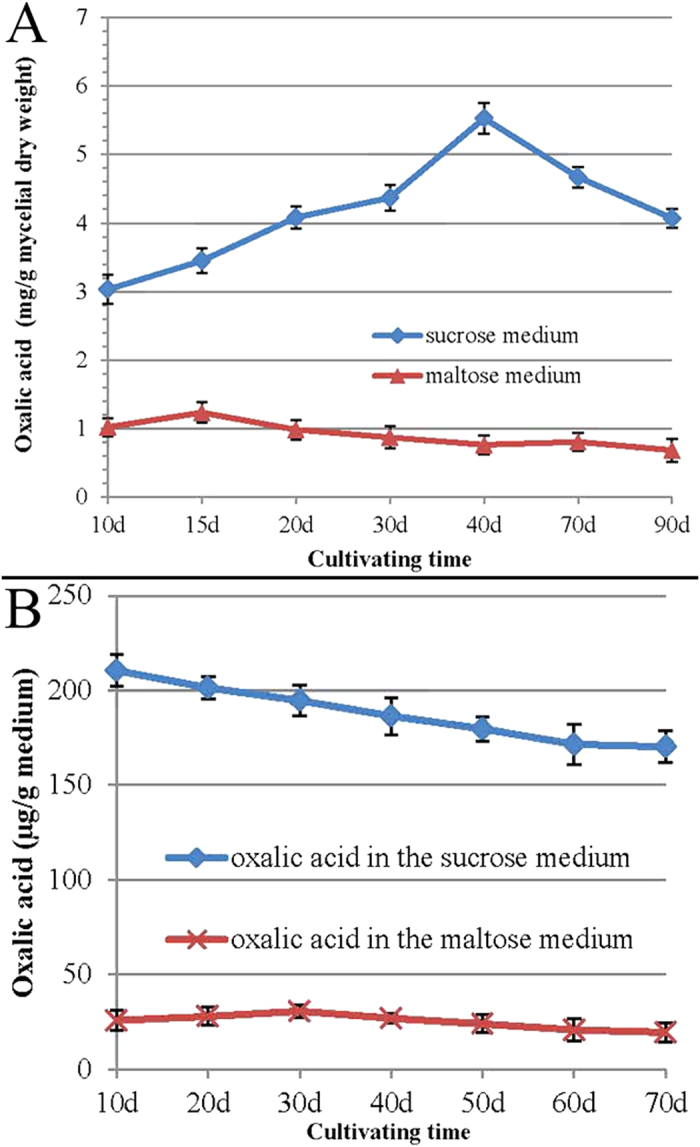
Determination of the endogenic oxalic acid of the mycelia and the medium. The oxalic acid concentration of the mycelia (**A**) and oxalic acid content of the maltose medium and the sucrose medium (**B**) were determined. The independent T-test was used to analyse the data of the two groups at each cultivation phase. Mean values ± SD or representatives of at least three independent experiments are shown, with 30 replicates in each group.

Mycelia differentiated and sclerotia appeared in the maltose medium, whereas no sclerotia appeared in the sucrose medium. The oxalic acid content of the mycelia in the sucrose medium was always higher than that in the maltose medium throughout the cultivation period (*p* < 0.05) (Fig. 3 panel A). The oxalic acid content increased with the passage of cultivation time, reaching 5.53 mg/mycelial dry weight at the 40^th^ day in the sucrose medium. This was 7.28-fold the concentration reached in the maltose medium. The oxalic acid content of the mycelia remained at low levels (below 1.5 mg/mycelial dry weight) in the maltose during cultivation (Fig. 3, panel A).

The oxalic acid content was much higher in the sucrose medium than in the maltose medium throughout the cultivation (*p* < 0.05) (Fig. 3 panel B). The oxalic acid concentration decreased gradually in the sucrose medium during cultivation, whereas it increased slightly and then decreased in the maltose medium during cultivation (Fig. 3 panel B).

**Figure 4:**
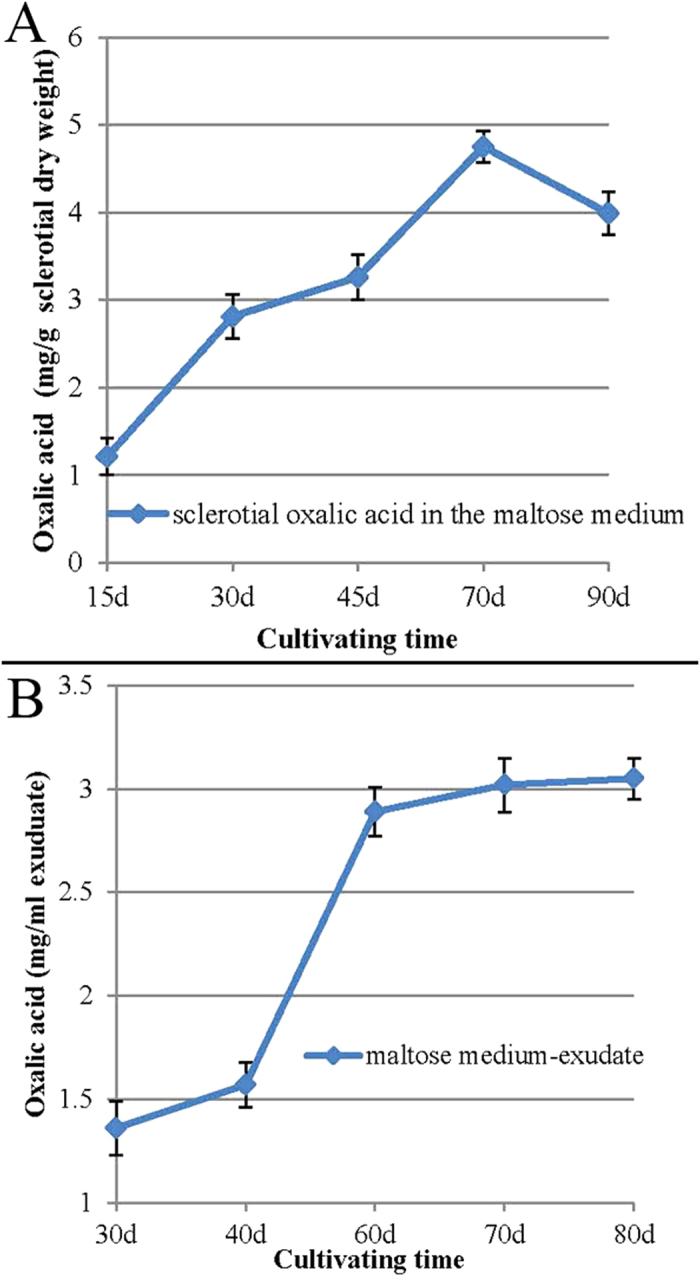
Determination of the endogenic oxalic acid of the sclerotia and the sclerotial exudate cultivated in the maltose medium. Oxalic acid content in the sclerotia (**A**) and the sclerotial exudate (**B**) in different stages of cultivation were determined. The data presented represent the means ± SD from three independent experiments, with 30 replicates in each group.

The oxalic acid concentration in the sclerotia induced by the maltose medium gradually increased from the SI stage (15 days after cultivation) to the SM stage (70 days after cultivation), ranging from 1.21 to 4.75 mg/g sclerotial dry weight. On the 90^th^ day after inoculation, it decreased a little (Fig. 4 panel A).

The oxalic acid content was high in the sclerotial exudate of the maltose medium, ranging from 1.36 to 3.05 mg /ml exudate during the cultivation time (Fig. 4 panel B). The oxalic acid levels increased sharply from the SD stage (30^th^ day of cultivation) to the SM stage of sclerotial formation (70^th^ day of cultivation), after which they were maintained at a relatively high level (Fig. 4 panel B).

## Discussion

It has been well documented that sclerotial differentiation in fungi is triggered by oxidative stress^31^,^32^,^33^. One recent study demonstrated that oxidative stress is involved in both sclerotial differentiation and aflatoxin B1 biosynthesis in *Aspergillus flavus*^34^. In the present study, the TBARS content in the mycelia of *P. umbellatus* cultivated in maltose medium with sclerotial formation was much higher than that cultivated in sucrose medium without sclerotial differetiation during cultivation (Fig. 1), which indicated that progress in sclerotial formation was accompanied by increased levels of lipid peroxidation products.

In the present study, exogenous oxalic acid was found to inhibit *P. umbellatus* sclerotial formation (Table 1), delaying sclerotial differentiation for at least 9 days and decreasing the TBARS content of the mycelia in a concentration-dependent manner (Fig. 2). This indicated that the exogenous oxalic acid may serve as a type of antioxidant during *P. umbellatus* mycelial differentiation. This result was in accordance with the finding of our previous study, in which we found that the antioxidant vitamin C inhibits *P. umbellatus* sclerotial formation and also reduces intracellular ROS generation in the mycelia^35^.

As our study showed, the oxalic acid accumulated at higher levels in the mycelia and surcrose medium than that of the maltose medium (Fig. 3 panel A; Fig. 3 panel B). No sclerotia could form in the surcose medium. At the same time, lipid peroxidation analysis indicated that the TBARS content of the mycelia during sclerotial formation in the maltose medium was much higher than in the sucrose medium (Fig. 1). Compared with the growth in the sucrose medium, the mycelia of *P. umbellatus* grown in maltose medium faced much more hyperoxidative stress, which induced the fungus to enter an oxidative stress state during sclerotial formation. On the contrary, it can be inferred that the lipid peroxidation products could not be accumulated to such an extent to form sclerotia in the sucrose medium, probably due to the high levels of oxalic acid inside. These findings imply that the endogenous oxalic acid might play an important role in reducing the oxidative stress in the mycelia and in the sucrose medium. As a result, it was difficult to trigger the generation of a hyperoxidant condition. We also tried using other culture mediums, such as fructose and glucose medium that could induce *P. umbellatus* sclerotial formation. In these culture media, similar results were obtained for the oxalic acid content and TBARS analysis in the medium, mycelia and sclerotia as that in the maltose medium(data not shown).

Droplet exudation (exudate), a common phenomenon in filamentous fungal mycelia and plants, is the active excretion of water and other dissolved materials. This often occurs on aerial parts of hyphae cultured on agar cultures. High amounts of nutrients could be reabsorbed, although oxalic acid could not be reabsorbed^36^,^37^. During sclerotial maturation, mycelial aggregation and hyphal coalescence may be facilitated due to the excretion of exudate^31^.

During *P. umbellatus* sclerotial formation, the fungus relies on antioxidant mechanisms to neutralize the excessive reactive oxygen species^26^. On one hand, the sclerotial exudate consists of fatty acids, ammonia, proteins and various enzymes, including the antioxidant enzymes peroxidase and catalase^31^. In the present study, the high content of oxalic acid in the sclerotial exudate in the maltose medium (Fig. 4 panel B) indicated that oxalic acid plays an important role as an effective antioxidant that alleviates oxidative stress during *P. umbellatus* sclerotial formation. On the other hand, it has been reported that enzymatic antioxidant defence systems, such as the superoxide dismutase (SOD) and the catalase (CAT) activities of the mycelia, increase during the SI stage and are maintained at high levels throughout the sclerotial differentiation process^16^.

Interestingly, the oxalic acid content of the mycelia (Fig. 3, panel A) and the maltose medium (Fig. 3, panel B) was low, whereas it accumulated at high levels in the sclerotia (Fig. 4, panel A) under the same cultivation conditions. This is probably because the *P. umbellatus* mycelia formed sclerotia to adapt to the hyperoxidant conditions by increasing the oxalic acid concentration and avoiding the oxidative stress state^26^,^38^. It is generally reasonable to assume that oxalic acid serves as an antioxidant during sclerotial differentiation.

Since ROS play important roles in diverse physiological processes^39^, the new insights into the relationship between oxidative stress and oxalic acid reported here might contribute to new knowledge of *P. umbellatus* sclerotial differentiation.

## Additional Information

**How to cite this article**: Xing, Y.-M. *et al*. Oxalic acid and sclerotial differentiation of *Polyporus umbellatus*. *Sci. Rep*. **5**, 10759; doi: 10.1038/srep10759 (2015).

## Figures and Tables

**Table 1:** The effects of exogenous oxalic acid on *P. umbellatus* sclerotial differentiation.

Exogenous oxalic acid (mg ml^−1^) (n = 30)	Time of SI appearance (day)	Sclerotial wet weight (g/20 g substrate)
0 (control)	15	1.61 ± 0.12
0.5	24	0.42 ± 0.09*
1.0	28	0.28 ± 0.08*
5.0		0
10.0		0

Values are presented as the mean ± SD for three independent experiments.

^*^p < 0.05 (compared to the control group).
